# Economy in Dental Practice

**Published:** 1897-08

**Authors:** R. E. Sparks

**Affiliations:** Kingston, Ont.


ARTICLE II.

### ECONOMY IN DENTAL PRACTICE.

BY R. E. SPARKS, D. D. S., KINGSTON, ONT.

Read before Eastern Ontario Dental Association, July 7th, '97.

The great social question of the day—the question which agitates the minds of all social reformers, and stares in the face all statesmen is, how shall the masses be fed? We can not close our eyes to the fact that this problem must soon be solved. The young men who rush into the profession thinking that, as far as they are concerned, they have solved it, will find some fine day that the question which had appeared as a molehill has become a mountain, that the question will include themselves. The rapid development which has taken place upon this continent in the last century has made a great demand for labor. That demand has made wages of all kinds high. But that development has about reached its high-water mark. Take, for instance, the tremendous amount of labor, manufacturing and business created by the building, equipped and operating of the net-work of railways and telegraph lines over the continent; to this add the thousands of villages and towns, and many of even large cities, which have sprung up during the time mentioned. To this again add the clearing up and bringing under cultivation of, and providing buildings for, the farms which provide food for the millions who have been attracted to this continent from the overflow of the old world. War with all its extravagance and devastation is, we hope, through the influence of Christian enlightenment largely a thing of the past. All these influences tend to lower the demand for men and increase the supply. This decrease of demand and increase of supply is bound to lower the scale of wages. This lowering of wages will apply to all classes, from the navvy who digs a ditch to the judge who sits on the bench. Labor organizations may fix a schedule of wages, professional societies may attempt to regulate the fees of their members, but remuneration for services will be governed not by the value of the services rendered so much as by the demand and supply of the servants; already we are feeling this in the dental profession. We see and hear discussed ways and means of excluding the hordes. This cannot be done. The lowering of remuneration for manual labor drives many into mercantile and professional pursuits. You may raise the standard of matriculation, lengthen the term of tuition, increase the college fees and stiffen the final examination. All these conditions will be met and fees will come down as in all the trades and professions. This is not a plea for a cheap dentistry, nor any apology for the lowering of fees before there be a necessity for it; neither is it an insinuation that we are too well paid for our services. It is only a prospect of what I consider an inevitable condition in the not very far future. How to meet it is a question of no small importance. The only way it can be met in my opinion is by economy. There is no doubt but that we on this continent are most extravagant livers, indulging in luxuries which the same class of Europeans would not dream of.

The one redeeming feature of the lowering of wages in competition is, it lowers the cost of production; so that while the purchasing power of the masses is reduced, the purchasing power of a dollar is increased. This is not so noticeable in the dental office as in the home. Still, where our material and instruments are expensive, the lessening of the cost becomes a factor in profits of a year's practice.

Combines may be formed, and every effort made to keep up prices of goods. They must come down. Already they are on the move. Price lists are being circulated with the announcement: "Reduced from —to

—" This reduction ranges from 6¼ per cent. as in the case of gold foils, to 33⅓ per cent. as in the case of some cements and alloys. Some of the manufacturers would fain have us believe that it is their magnanimity being manifested, that it has merely been a question of how soon and how much. As we have watched them corner the platiuum market and advance the price of teeth, form combines to keep up prices of goods, it has appeared to us that it was rather a question of how long and how little. But leaving the necessity of economy out of the question it may be profitable to consider the subject as a matter of policy. While few of us expect to amass wealth from the practice of our profession, we all hope to lay up a little for a rainy day, or for that time which must come to us all, when the eye grows dim and the hand begins to lose its cunning, when we begin to see our patients leave us for our younger and more active confreres.

In no case more than in the dental office and laboratory is realized the truisms, "wilful waste brings woful want," and "economy is the sure road to wealth." It is not the amount a man earns as much as the amount he saves that governs his financial standing. Granted that economy is desirable in dental practice at all times, the question arises, how can we economize? First, in the selection of an office. It is desirable to have an office in a respectable part of the town or city. But there are in every city locations where office rents are nearly double the rent for the same accommodation in any other part, which, for dental purposes, may be just as desirable. Then the selection of an office governs the saving or waste of many things that are valuable to a dentist. The rooms should be compact so they may be more easily heated; for the fuel bill in a Canadian winter is no small item. Besides compactness and convenience of arrangement saves time and strength, both of which are important items in the dentist's stock-in-trade. The office should be well lighted. The fine work in an obscure location makes no ordinary strain upon the eyesight under the most favorable circumstances, but to operate in a poorly lighted office is an imposition which no ordinary eyes will tolerate for many years. The best light can generally be secured in upstairs rooms. The office should be away from the residence to avoid having to work at unseasonable hours.

We should practice economy of strength. The dental profession is considered to be one of the most unhealthful of occupations. Close confinement, close application, breathing the breath of patients, and in many cases of those diseased in lungs or schneiderian membrane. In many cases, long hours operating during the day time and doing laboratory work or attending to books and correspondence at night; all this tends to bring on indigestion, constipation, hæmorrhoids, headache and neuralgia from overworked eyes and brain. The laboratory should be close to the operating room to save unnecessary steps. In no case should the laboratory be on a different flat from the operating room. As the basement kitchen is an abomination to a residence, so is a dental laboratory above or below an operating room.

The arrangement of a dental office and the care of a dentist's health would make fruitful subjects for lengthy papers. Therefore we cannot more than mention them here; the object of this paper being more directed to the little wastes which may take place in a dental practice. Time may be saved by carrying the work along so as not to have to wait for anything; for instance, if two impressions have to be taken for the same individual, take one and run it. The cast will be hardening while the second impression is being taken and run. The second will harden while the first is being taken from the impression, and an articulating plate fitted. When the articulation is taken run it and it will harden while the teeth are being selected.

Having ground and articulated the teeth, wax and invest one set. By the time the second set is waxed and invested the first will be ready to wash out and pack; when this is done the second will be ready and may be washed out and packed while the first is heating preparatory to being closed; while they are vulcanizing other work may be gone on with.

Much material may be wasted. It is an easy matter to mix twice as much plaster as is needed for an impression or investment. A case may be waxed much thicker and covering more space than is required for the plate, making a waste of rubber and time in finishing up. Much gold may be wasted in finishing gold plates. crowns or bridge-work. Often much more solder is used than is necessary for the strength or finish of the piece. In finishing gold work, if dry corrundum or carborundum stones are used while grinding down the solder and the piece held over a paper or other receptacle, or if wet stones are preferred, or bowl containing water be used to wet the stone and wash off the piece occasionally, one will be surprised at the accumulation of grindings. The fine sand-paper or emery cloth used for rubbing down gold work and the strips and disks used in finishing gold fillings should be saved and burned and the ashes preserved. Sheet wax may be easily and economically made. Quite a saving of gas may be made by watching carefully the heaters and waxing burners. It is quite possible to make the gas bill what it need be. Great waste may be made in the careless mixing of cement and amalgam. One not infrequently sees more cement remaining upon the mixing slab, or more amalgam upon the bracket, after the operation than was used in the fillings themselves. All amalgam scrap should be saved, as it is easily refined and recut. Sweepings of the operating room and laboratory could profitably be kept, as they are readily bought by the refiners. I have heard of old carpets off operating rooms being sold for more than enough to purchase new ones. I have never been able to negotiate an exchange so favorably. Possibly because I have my carpet beaten twice a year; but I have often intended having it rolled up and opened out upside down upon sheets and so beaten, to preserve the gold finished off fillings; I feel that it would be a paying precaution. Rubber dam which has become too full of holes to make it safe to use, may be made as good as ever by patching the holes with a little of itself rubber cement. Towels used for protecting the patient's clothing are worn out more by laundrying than by use. They may be used much longer without being laundered if, instead of wiping our instruments upon them while operating, we wipe them upon a mouth napkin, which answers every purpose and which costs relatively nothing. In the use of nerve broaches we may be extravagant. We may buy them at $3 or less per gross, or we may pay $7 or $8 for the same number. With care the one grade will answer about as well as the other. But whatever grade we use we may destroy many more than are necessary. In ordinary cases one broach will remove several nerves before the barks are stripped off, if it be only used for the removal of the nerves. As soon as it has done its work it should be cleansed and laid away for another time, and others which have lost their usefulness as extirpators may be used for washing out the canals, and when they have become so smooth as to refuse to hold a twist of cotton, they may have their points snipped off and be further used for nerve canal pluggers.

That pet ligature, floss silk, may be replaced by gilling twine, of which we may buy as much for 50c. as of the former for $5. When using a ligature it is usually required upon three or more teeth. We are directed to prepare one for each and to tie a knot to go on palatal or lingual side of the tooth to prevent the rubber from slipping off. Prepare one ligature; knot it if you wish, tie it on one tooth, cut off the ends close to the tooth, tie the ends together and proceed with as many teeth as it is necessary to ligate. It is profitable to purchase time-saving instruments and devices; but let us not buy everything that is offered for sale or we will find our laboratory and closet shelves loaded with rubbish only to turn up at house-cleaning time. Thus by a little care and attention all the way along the line, and we have only taken time to mention a few representative points, we may make the expenses of the dental practice about one-half what it may be by carelcss-ness and extravagance.—*Dominion Dental Journal.*

